# Cannabis use and risk of schizophrenia: a Mendelian randomization study

**DOI:** 10.1038/mp.2016.252

**Published:** 2017-01-24

**Authors:** J Vaucher, B J Keating, A M Lasserre, W Gan, D M Lyall, J Ward, D J Smith, J P Pell, N Sattar, G Paré, M V Holmes

**Affiliations:** 1 Department of Internal Medicine, University Hospital of Lausanne, Lausanne, Switzerland; 2 Department of Surgery, University of Pennsylvania, Philadelphia, PA, USA; 3 Centre for Psychiatric Epidemiology and Psychopathology (CEPP), University Hospital of Lausanne, Prilly, Switzerland; 4 Oxford Centre for Diabetes, Endocrinology, and Metabolism, Churchill Hospital Campus, University of Oxford, Oxford, UK; 5 Wellcome Trust Centre for Human Genetics, University of Oxford, Oxford, UK; 6 Institute of Health & Wellbeing, University of Glasgow, Glasgow, UK; 7 Institute of Cardiovascular and Medical Science, University of Glasgow, Glasgow, UK; 8 Population Health Research Institute, Hamilton Health Sciences, Department of Clinical Epidemiology and Biostatistics, McMaster University, Hamilton, ON, Canada; 9 Population Genomics Program, Chanchlani Research Centre, McMaster University, Hamilton, ON, Canada; 10 Department of Pathology and Molecular Medicine, McMaster University, Hamilton, ON, Canada; 11 Thrombosis and Atherosclerosis Research Institute, McMaster University, Hamilton, ON, Canada; 12 Clinical Trial Service Unit & Epidemiological Studies Unit (CTSU), Nuffield Department of Population Health, University of Oxford, Oxford, UK; 13 Medical Research Council Population Health Research Unit at the University of Oxford, Oxford, UK

## Abstract

Cannabis use is observationally associated with an increased risk of schizophrenia, but whether the relationship is causal is not known. Using a genetic approach, we took 10 independent genetic variants previously identified to associate with cannabis use in 32 330 individuals to determine the nature of the association between cannabis use and risk of schizophrenia. Genetic variants were employed as instruments to recapitulate a randomized controlled trial involving two groups (cannabis users vs nonusers) to estimate the causal effect of cannabis use on risk of schizophrenia in 34 241 cases and 45 604 controls from predominantly European descent. Genetically-derived estimates were compared with a meta-analysis of observational studies reporting ever use of cannabis and risk of schizophrenia or related disorders. Based on the genetic approach, use of cannabis was associated with increased risk of schizophrenia (odds ratio (OR) of schizophrenia for users vs nonusers of cannabis: 1.37; 95% confidence interval (CI), 1.09–1.67; *P*-value=0.007). The corresponding estimate from observational analysis was 1.43 (95% CI, 1.19–1.67; *P*-value for heterogeneity =0.76). The genetic markers did not show evidence of pleiotropic effects and accounting for tobacco exposure did not alter the association (OR of schizophrenia for users vs nonusers of cannabis, adjusted for ever vs never smoker: 1.41; 95% CI, 1.09–1.83). This adds to the substantial evidence base that has previously identified cannabis use to associate with increased risk of schizophrenia, by suggesting that the relationship is causal. Such robust evidence may inform public health messages about cannabis use, especially regarding its potential mental health consequences.

## Introduction

Cannabis is the most widely misused illicit drug with an estimated 182 million consumers in 2013 globally.^1^ Several high-profile observational studies have reported a positive, dose-dependent association between cannabis use and risk of schizophrenia, especially in young people in whom cannabis use is particularly high.^2^ The lifetime risk of schizophrenia is ∼0.7%, and the natural history of disease carries a high risk of long-term symptoms and disability together with a reduced life expectancy.^3^ In addition, schizophrenia represents a high economic burden with an estimated cost of $63 billion per year in the United States.^4^ Clarifying the causal role between cannabis use and risk of schizophrenia is therefore important to understanding the health impacts of cannabis exposure and to inform on potential preventative strategies to alleviate the burden of disease from schizophrenia.^5^

A substantial body of observational evidence supports the hypothesis that cannabinoids play a role in the development of schizophrenia.^2^ Prospective observational studies, with decades of follow-up and accounting for a large number of potential confounding factors (such as demographic, family history, personal history, socioeconomic or other environmental markers) have consistently demonstrated that exposure to cannabis is associated with an increased risk of schizophrenia or related disorders.^2^ These findings have been reinforced by basic research experiments that point to cannabis altering various neurotransmission pathways linked to pathogenesis of psychotic disorders and by interfering with neurodevelopment in adolescents.^6^ Despite this, any causal link between cannabis use and psychotic disorders remains controversial as observational findings can always be hampered by confounding (where another risk factor associated with cannabis actually causes the disease) and/or reverse causality bias (where individuals affected by schizophrenia may be more prone to consume cannabis).^2, 7^ Moreover, cannabis use is strongly associated with tobacco consumption and the latter has been observationally related to risk of schizophrenia meaning smoking could confound the link between cannabis and schizophrenia.^8^

In the setting where a randomized trial—representing the optimal method to test a clinical hypothesis—of a harmful exposure (such as cannabis consumption) would be unethical, a genetic approach represents a valid alternative to assess causality free from confounding or reverse causality bias.^9^ Using Mendelian randomization (MR) principles, causality between an exposure (such as cannabis use) and an outcome (for example, schizophrenia) can be tested through use of genetic markers that associate with the exposure, employed as instruments providing certain assumptions are met.^10^ Recent developments of MR facilitate assessing the robustness of the causal effect estimate by testing for presence of pleiotropy (where genetic markers associate with the outcome through more than one causal pathway, also known as horizontal pleiotropy). Egger Mendelian randomization (MR-Egger) and weighted median MR provide statistical tests for presence of pleiotropic effects of the single-nucleotide polymorphisms (SNPs) under analysis (and provide a causal estimate that takes this into account).^11, 12^ On the other hand, multivariable MR provides a causal estimate for an exposure that statistically adjusts for potential pleiotropic effects of the genetic marker(s) with a risk factor (for example, tobacco consumption).^13^

We used SNPs associated with ever use of cannabis reported in a recent genome-wide association study (GWAS)^14^ as instruments to clarify the causal role of cannabis consumption on risk of schizophrenia. We then assessed for presence of pleiotropy of the genetic markers through MR-Egger and weighted median MR. We further adjusted for potential shared pathways with tobacco consumption in multivariable MR. We additionally conducted sensitivity analyses by restricting to SNPs with putative functional roles and by sequentially excluding each SNP from the analysis. Finally, we compared the causal estimate with a meta-analysis of observational studies.

## Materials and methods

### Observational analysis between ever use of cannabis and risk of schizophrenia

Observational studies reporting an association between cannabis use and risk of schizophrenia were selected from a recent and comprehensive review of the literature (published in 2016) and a meta-analysis from 2007 reporting prospective studies of cannabis use and risk of schizophrenia.^2, 15^ As only one study reported schizophrenia as an outcome,^16^ we slightly broadened our inclusion criteria to also include studies reporting related disorders (schizophreniform disorder and psychotic symptoms). To identify additional studies that may be eligible for inclusion since the meta-analysis from 2007, we conducted a PubMed search (Supplementary Figure S1).

To compare with the causal estimate (see below), we restricted to studies that reported ever use of cannabis (compared with never users of cannabis) as an exposure and a corresponding risk estimate for schizophrenia or related disorders. We identified four studies that met these criteria.^16, 17, 18, 19^ We found one additional study in which the definition of the exposure was similar (any use of cannabis, provided that individuals have consumed cannabis ⩾5 times) and also included it in the analysis.^20^ The pooled effect estimate was derived using a random-effects meta-analysis of study summary estimates. Supplementary Tables S1 and S2 summarize the main characteristics of included and excluded studies, respectively.

### Genetic markers associated with ever use of cannabis

We used the 10 leading SNPs from a recent GWAS (contributing studies outlined in Supplementary Table S3), comprising data of participants from European ancestry predominantly, on cannabis use (phenotype defined as ever use of cannabis during participants’ lifetime) to obtain the gene–exposure (SNP–cannabis) association estimates and their corresponding standard errors (s.e.) values (Supplementary Table S4).^14^ Although none of the SNPs surpassed a conventional genome-wide significance threshold (*P*-values were between 4.6 × 10^−7^ and 3.1 × 10^−6^ in the discovery analysis), estimates were directionally consistent across the vast majority of contributing studies (Supplementary Table S4), and these SNPs can individually, and cumulatively, be considered as valid instruments for MR analysis.^21^

### Association between cannabis-associated genetic markers and risk of schizophrenia

The gene–outcome (SNP–risk of schizophrenia) association estimates were obtained using the publicly available GWAS repository on schizophrenia from the Psychiatric Genomics Consortium (http://www.med.unc.edu/pgc/downloads). Supplementary Table S5 describes the contributing studies. SNPs were directly matched with the 10 SNPs associated with ever use of cannabis. The number of individuals and the relationships between datasets are presented in Supplementary Figure S2. We used the same reference allele for each SNP to orientate cannabis and schizophrenia estimates.

### Statistical analysis

MR analysis was conducted by first generating an instrumental variable estimate for each SNP. The instrumental variable estimate for each SNP was generated by dividing the association of each SNP with risk of schizophrenia by the corresponding association with risk of ever use of cannabis and the s.e. was estimated using the delta method.^22^ We pooled instrumental variable estimates across SNPs using fixed-effect (inverse variance weighted) meta-analysis. As a sensitivity analysis we also pooled estimates using random-effects modelling. Estimates of the association of each SNP with ever use of cannabis were not transformed. In order to generate a MR estimate for ‘users vs non-users’ of cannabis (as opposed to a per-1-log unit increase in ever use of cannabis), we transformed the summary estimate from meta-analysis using estimates of risk of schizophrenia in the population, and the prevalence of schizophrenia in never users of cannabis, as previously described.^23^ A full description of the methodology is provided in the Supplementary Information.

### Characteristics of the genetic markers

#### Strength of instrument and power to detect a causal effect

In MR analyses, but especially in the context where multiple SNPs that did not achieve GWAS significance are used cumulatively, there are certain characteristics that need to be tested.

First, a concern might be weak instrument bias. Conventionally, when using data sets that overlap for the SNP–exposure and SNP–outcome, this can generate biased estimates and yield a spurious causal estimate (arising from correlation of the error terms of SNP–exposure and SNP–outcome).^24^ However, in our case, there was only minimal overlap (<5%) between the data sets used to derive the effect estimates for SNPs with ever use of cannabis and risk of schizophrenia (Supplementary Figure S2), minimizing the possibility of weak instrument bias yielding a false positive association. In contrast to overlapping datasets where weak instrument bias can lead to a false positive result, use of *non-overlapping data sets* in MR can lead to a false negative association.^24^ This means that a positive result from a MR analysis when using non-overlapping data sets protected from such bias.

We estimated instrument strength by calculating the proportion of variance in use of cannabis explained by each SNP. We then derived the F-statistic of each SNP individually and cumulatively (full details provided in the Supplementary Information).

We estimated power to detect the same magnitude of association reported in the observational studies, using a two-sided α of 0.05. Power was 100% and is presented in Supplementary Table S6.

#### Assessment of directional pleiotropy

We tested for presence of unmeasured pleiotropy of the genetic markers using MR-Egger as described by Bowden *et al.*^11^ Essentially, this uses the same principles of testing for small study bias in meta-analysis. The methodology was similar as for conventional MR analysis (described above), with the exception that all alleles (and corresponding estimates) were oriented in the direction of an increase in the exposure before the analyses. The s.e. was obtained by bootstrap resampling 10 000 times. As a sensitivity analysis, we measured the relative bias in the MR-Egger causal effect estimate because of the variance of the estimates of the SNP–cannabis association.^25^ Indeed, all MR analyses rely on the assumption that the SNP–exposure association is true (NO Measurement Error (NOME) assumption),^25^ but whenever the SNP–exposure association estimates are imprecise, weak instrument bias can distort the causal effect estimate. The *I*^2^ statistic, quantifying weak instrument bias in the context of MR-Egger, was moderate (*I*^2^=67% potential bias of 43%). As described by Bowden *et al.*,^25^ we then applied simulation extrapolation (implemented in R using the *simex* package) to adjust the MR-Egger causal estimates to account for a potential NOME violation.

We also conducted a penalized weighted median MR analysis (implemented in Stata using the mrrobust package; available at: https://github.com/remlapmot/mrrobust). This approach gives more weight to genetic variants with homogeneous causal estimates (that is, close to the median causal estimate) even when up to 50% of the weight in the analysis arises from invalid genetic markers.^12^

### Sensitivity analyses

As tobacco consumption has been related to risk of schizophrenia and use of tobacco shares a strong genetic correlation with use of cannabis in Stringer *et al.*,^8, 14^ we conducted a multivariable MR—to adjust for shared pathways with and/or potential confounding by tobacco—using summary statistics for the association of each of the 10 cannabis-related SNPs with tobacco (ever vs never smokers) derived from 111 898 participants (51 984 ever smokers and 59 914 never smokers) from the UK Biobank (http://www.ukbiobank.ac.uk). Selection of participants and genotyping are described in the Supplementary Information. Multivariable MR was conducted by regressing the SNP–cannabis estimates on SNP–schizophrenia estimates adjusting for SNP–tobacco estimates.^13^ The s.e. was obtained by bootstrap resampling 10 000 times.

We conducted two additional sensitivity analyses. First, we assessed the robustness of the summary causal estimate to influence by individual SNPs. This was done by: (1) sequentially removing each SNP from the MR analysis (leave-one-out permutation analysis); (2) estimating studentized residuals to assess whether any individual causal estimate was an outlier (as proposed by Corbin *et al.*^26^); and (3) computing Cook’s distance to identify influential SNPs on the overall model.

Second, we restricted the analyses to two SNPs (rs73067624 and rs4471463) located within two genes (*KCNT2* (1q31) and *NCAM1* (11q23), respectively) that were associated with ever use of cannabis in the gene-based tests of associations in Stringer *et al.*^14^ These two genes are potentially functional: *KCNT2* encodes a potassium voltage-gated channel that may play a role in addiction.^14, 27^ Previous studies have found that markers linked to *KCNT2* are related to cocaine dependence and opioid consumption.^27^ *NCAM1* regulates pituitary growth hormone secretion and is implicated in dopaminergic neurotransmission,^14^ and has been associated with dependence to nicotine, alcohol and heroin.^28^

All statistical analyses were conducted using Stata v.13.1 (Stata, College Station, TX, USA), except computation of *I*^2^ statistic and simulation extrapolation analyses that were conducted using the statistical programme R (version 3.3.1).

## Results

### Observational association between ever use of cannabis and risk of schizophrenia and related disorders

**Figure 1:**
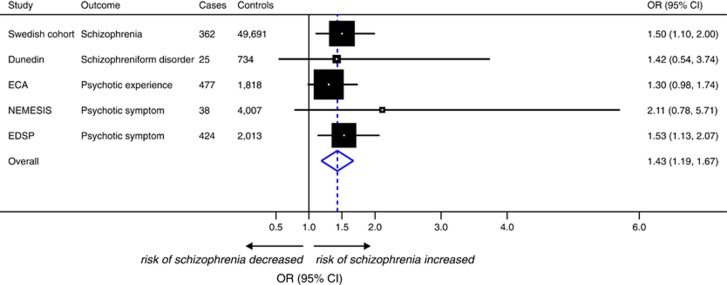
Meta-analysis of prospective observational studies reporting an association between use of cannabis and risk of schizophrenia or related disorders. Meta-analysis uses a random-effects model. Studies are sorted by type of outcome (schizophrenia only vs schizophrenia and related outcomes). Odds ratios (ORs) and 95% confidence intervals (CIs) express the risk of schizophrenia or psychotic symptoms for ever use of cannabis (compared with never use). For additional information on each study, see Supplementary Table S1. Dunedin, Dunedin Multidisciplinary Health & Development Study; ECA, Epidemiologic Catchment Area; EDSP, Early Developmental Stages of Psychopathology Study; NEMESIS, Netherlands Mental Health Survey and Incidence Study; SC, Swedish Cohort.

One prospective study met our primary research criteria and reported that ever use of cannabis (compared with no use) was associated with an odds ratio (OR) for schizophrenia of 1.50 (95% confidence interval (CI), 1.10–2.00). When meta-analysing this estimate with other prospective observational studies reporting related traits, including schizophreniform disorder and psychotic symptoms (encompassing a total of 1326 cases and 58 263 controls), ever use of cannabis was associated with a 43% increase in the risk of schizophrenia or related disorders (OR, 1.43; 95% CI, 1.19–1.67; *I*^2^=0%) using random-effects modelling (Figure 1).

### Causal effect of ever use of cannabis on risk of schizophrenia

**Figure 2:**
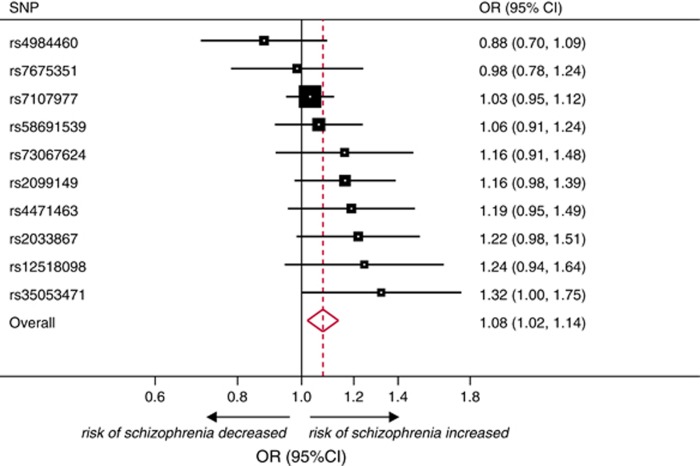
Meta-analysis of the association of genetically instrumented use of cannabis and risk of schizophrenia for the 10 single-nucleotide polymorphisms (SNPs) under analysis. Odds ratios (ORs) and 95% confidence intervals (CIs) express the risk of schizophrenia per-1-log unit increase in ever use of cannabis. Meta-analysis uses a fixed effect model. The method to derive the population-based OR of schizophrenia among users of cannabis compared with nonusers (OR 1.37; 95% CI, 1.09–1.67), as presented in the main text and Figure 3, is described in the Supplementary Information.

**Figure 3:**
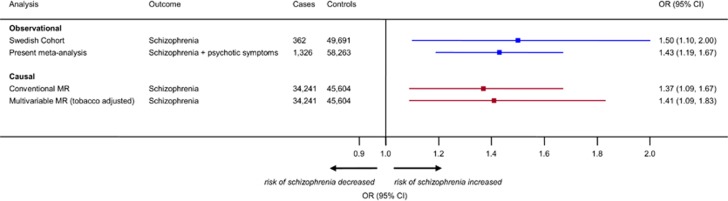
Comparison of observational (blue) and causal (red) estimates for use of cannabis and risk of schizophrenia. Two observational estimates are provided according to a stringent definition of schizophrenia (as reported in the Swedish cohort^16^) or to an outcome comprising studies reporting risk of schizophrenia or psychotic symptoms (derived from the meta-analysis reported in Figure 1) for ever use of cannabis. Causal estimates represent population-based associations derived by conventional (Figure 2) and multivariable Mendelian randomization (MR). The total number of cases and controls in each analysis are presented.

The 10 SNPs associated with ever use of cannabis explained 1% of its variance. There was a positive estimated effect between ever use of cannabis and risk of schizophrenia (Supplementary Figure S3). In MR analysis based on 34 241 cases of schizophrenia and 45 604 controls, ever use of cannabis was causally associated with risk of schizophrenia (OR per-1-log unit increase in ever use of cannabis (derived by fixed-effect meta-analysis of individual causal effects estimates of the SNPs), 1.08; 95% CI, 1.02–1.14; *P*-value=0.007; Figure 2). Random-effects meta-analysis yielded similar results (OR per-1-log unit increase in ever use of cannabis, 1.09; 95% CI, 1.02–1.16; *P*-value=0.010). Applying population-based estimates, this translated to a 37% increase in the risk of schizophrenia (OR for users vs non-users of cannabis, 1.37; 95% CI, 1.09–1.67; Figure 3). The MR estimate was consistent with estimates derived from observational analyses restricted to schizophrenia alone (test for heterogeneity, χ^2^=0.23; *P*-value=0.634) or schizophrenia and related disorders combined (test for heterogeneity, χ^2^=0.10; *P*-value=0.755; Figure 3).

### Assessment of pleiotropic effects of the genetic markers

We did not find evidence against the null hypothesis of no unmeasured pleiotropy of the genetic markers using MR-Egger (*P*-value for pleiotropy=0.292). The estimate derived from MR-Egger is compared with conventional MR estimates in Supplementary Figures S4 and S5. Compared with conventional MR results, weighted median MR produced very similar causal estimate although with reduced precision. Supplementary Table S7 contrasts conventional MR, MR-Egger, MR-Egger adjusted for simulation extrapolation and weighted median MR causal effect estimates.

Adjusting for the association of SNPs for smoking in multivariable MR did not show evidence of shared pathways and/or confounding with a causal effect estimate of schizophrenia from users of cannabis that remained stable (OR, 1.41; 95% CI, 1.09–1.83; Figure 3).

### Sensitivity analyses

**Figure 4:**
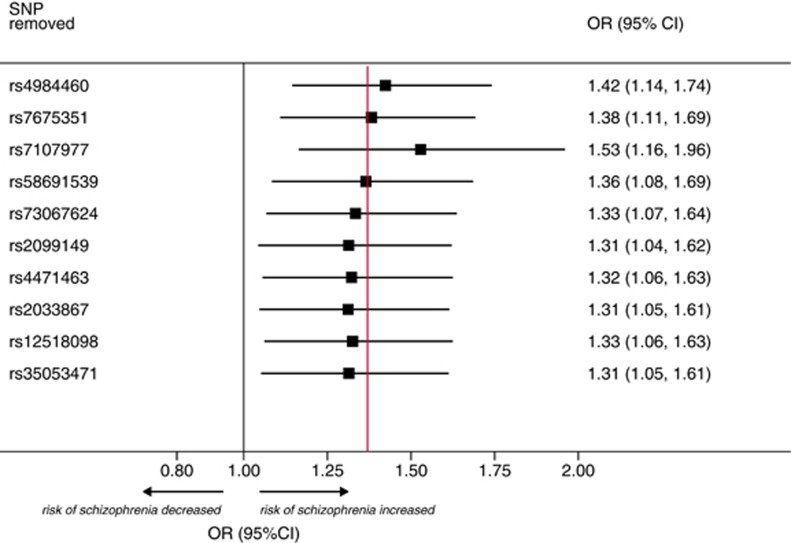
Sensitivity analysis of the association of use of cannabis and risk of schizophrenia by sequentially removing each single-nucleotide polymorphism (SNP) from the analysis. The red vertical line represents the summary causal effect estimate (derived from Mendelian randomization) when including the 10 SNPs in the analysis (presented in Figure 3). Odds ratios (ORs) and 95% confidence intervals (CIs) represent the population-based risk of schizophrenia in users of cannabis (compared with nonusers).

To further test the stability of the MR effect estimate to inclusion of SNPs that could individually distort the genetic association between cannabis use and schizophrenia, we sequentially removed each SNP from the analysis. The direction and precision of the summary association between ever use of cannabis and risk of schizophrenia remained largely unchanged using this approach (Figure 4). None of the individual estimates for each SNP was an outlier using studentized residuals (Supplementary Figure S6) and Cook’s distance showed that only two SNPs (rs2033867 and rs7107977) had marginal influence level on the overall model (Supplementary Figures S7 and S8). Furthermore, restricting the analysis to two putative functional SNPs (rs73067624 and rs4471463) showed a persistent causal effect (OR for users vs nonusers of cannabis, 1.88; 95% CI, 1.00–3.21; Supplementary Figure S9).

## Discussion

Using a genetic approach, this study provides additional evidence that cannabis use is likely to increase the risk of schizophrenia. This finding corroborates many previous prospective observational studies that identified cannabis users to be at increased risk of schizophrenia. As cannabis is the leading drug of misuse, this finding is timely to draw attention to the potential mental health consequences of cannabis use and to provide more robust scientific evidence to inform the public health debate on cannabis legalization.

During the past 30 years, epidemiological observations have consistently demonstrated a strong, positive and dose-dependent association between cannabis use and risk of psychotic disorders.^2, 15^ The direction and the strength of the association persisted after adjusting for measured confounders and with long periods (∼25 years) of follow-up (to attempt to minimize confounding and reverse causality bias, respectively). Our meta-analysis of prospective observational studies confirmed these findings in a magnitude that tallies remarkably closely with previous reports.^15^ Despite the consistency of observational data, clarifying whether or not cannabis use causally influences risk of schizophrenia has remained challenging. This is because observational studies, even accounting for confounding factors, can be affected by biases that undermine the validity (such as residual confounding).^2^ As such, the ability to answer the question on causality has been at an impasse, as a randomized controlled trial (considered the gold standard to test a hypothesis) is not possible for ethical reasons, as it would involve exposing participants to a potentially harmful exposure (a similar scenario to examining whether alcohol protects against risk of cardiovascular disease).^29^ In this setting, an MR approach can provide pivotal information on causality that can be of public health importance and inform guidelines.^30^ Our findings strongly support the large body of evidence from observational studies that exposure to cannabis plays a causal role in the development of schizophrenia.

Our findings are supported by studies that show that expression of schizophrenia-associated cerebral cannabinoid receptors are modified by cannabis use^31^ and that cortical maturation is altered by cannabis use in adolescents.^32^ More compellingly, small randomized trials involving human participants in laboratory conditions suggest that exposure to delta-9-tetrahydrocannabinol confers a risk to developing symptoms that mimic psychotic disorders.^6^ Observationally and genetically, tobacco use is strongly correlated with cannabis use and has been proposed to act synergistically with cannabis to establish addiction.^8^ Moreover, the association between cannabis and psychotic experiences has been shown to be influenced by tobacco use, that is, accounting for tobacco use reduces the cannabis–schizophrenia relationship.^33^ Hence, the lack of attenuation in the MR estimate when adjusting for by tobacco consumption, as tested in our multivariable MR analysis, strengthens the findings of a primary association between cannabis use and risk of schizophrenia. Finally, our sensitivity analysis restricting to two genes with presumptive functional roles in drug dependence may suggest that cannabis affects addiction mechanisms that in turn influence the risk of schizophrenia. However, against this theory is the observation that other drugs of addiction are less associated to risk of schizophrenia or related disorders.^34^ Moreover, any influence of addictive mechanisms would not undermine our findings, as cannabis exposure may be necessary to establish dependence, and addiction mechanisms could lie on the same causal pathway (Supplementary Figure S10).

Limitations include that our study did not permit investigation of the risk of schizophrenia in relation to the quantity, type, route of administration or indeed the age at exposure to cannabis. Second, the precise mechanisms explaining how some of the genetic markers under analysis alter cannabis use (or dependence) remain unknown; however, this is not a necessary requirement to conduct a MR analysis using multiple loci. Third, the SNPs used in the analysis did not reach conventional genome-wide association significance thresholds. However, directions of effect in the discovery GWAS were consistent in the vast majority of contributing studies (Supplementary Table S4) and combining individual SNPs for an analysis such as this remains valid provided the genetic instrument does not suffer from weak instrument bias. In that regard, in the context of conducting summary-level MR analysis using non-overlapping data sources for the exposure and outcome (as we report here), weak instrument bias would bias the effect towards the null (that is, opposite to weak instrument bias in overlapping data sets).^24^ This greatly increases confidence in the causal effect estimate that we report. Furthermore, our sensitivity analyses identified that the causal estimates from MR were robust to various approaches to test for stability of the causal estimates. Fourth, MR-Egger and weighted median MR may have been underpowered to detect directional pleiotropy of the genetic instruments (if it were present).^11^ Nevertheless our sensitivity analyses testing the influence (including any pleiotropic effect) of any individual SNP, based on the leave-one-out permutation analysis (Figure 4) and excluding two SNPs with potential influence on the overall model (Supplementary Figure S8), showed that the causal estimate remained robust. It is noteworthy that, despite these potential limitations, this study represents the closest approximation to a randomized trial on the effect of ever use of cannabis and risk of schizophrenia.

In summary, a genetic approach—representing an alternative to assessing causality when a randomized controlled trial would be unethical—strongly supports the hypothesis that use of cannabis is causally related to risk of schizophrenia. This may help inform public health debate on cannabis use and preventive strategies to alleviate the burden of disease from schizophrenia.
