# Supplementary material for: Cannabis use and risk of schizophrenia: a Mendelian randomization study
Source: Mol Psychiatry. 2017 Jan 24;23(5):1287–92. doi: 10.1038/mp.2016.252 (PMC5984096; doi:10.1038/mp.2016.252)
Supplement: Supplementary Information [file mp2016252x1.docx]

**Supplement**

**Cannabis use and risk of schizophrenia: a Mendelian randomization analysis**

**submission after 2nd revision**

by Julien Vaucher, MD, Brendan J. Keating, PhD, Aurélie M. Lasserre, MD, Wei Gan, PhD, Donald M. Lyall^6^, PhD, Joey Ward^6^, BSc, Daniel J. Smith^6^, MD, Jill P. Pell^6^, MD, Naveed Sattar^7^, MD, PhD, Guillaume Paré, MD, Michael V. Holmes, MD, PhD

**Table of Contents**

**Supplementary analysis pp.**

The population-based risk of schizophrenia among users compared to non-users

of cannabis 3-4

Proportion of variance in use of cannabis 5

UK Biobank: general description, genotyping, selection of participants and statistical analysis 6-7

**Supplementary Tables**

**Table S1** Summary of the observational studies included in the analysis of cannabis

use and risk of schizophrenia, presented by year of recruitment 8

**Table S2** Summary of the observational studies excluded from the analysis of

cannabis use and risk of schizophrenia with reasons for exclusion,

presented by year of recruitment 9

**Table S3** Studies included in the cannabis-GWAS 10

**Table S4** Summary of the 10 SNPs associated with use of cannabis 11

**Table S5** Studies included in the Schizophrenia-GWAS 12

**Table S6** Power (two-sided α=0.05) for conventional Mendelian randomization

analysis 13

**Table S7** Comparison of conventional MR, MR-Egger, MR-Egger adjusted for SIMEX

and weighted median MR causal effect estimates of cannabis use on risk

of schizophrenia 14

**Supplementary Figures**

**Figure S1** Flowchart of observational meta-analysis 15

**Figure S2** Venn diagram showing the number of individuals and the overlap

between the cannabis-GWAS and the schizophrenia-GWAS 16

**Figure S3** Pair-wise association plot of the 10 SNPs associated with cannabis use

and risk of schizophrenia 17

**Figure S4** Scatter plot of the genetic association with cannabis use against

genetic association with schizophrenia 18

**Figure S5** Funnel plot of the instrument strength (minor allele frequency corrected genetic association with cannabis use) against causal estimates

of cannabis use on schizophrenia 19

**Figure S6** Studentized residuals of conventional MR analysis 20

**Figure S7** Cook’s distance of conventional MR analysis 21

**Figure S8** Contrasting main results with analysis removing two SNPs with potential influential level on the model based on Cook’s distance 22

**Figure S9** Sensitivity analyses of the association of cannabis use and risk of

schizophrenia restricting to two SNPs with putative functional roles 23

**Figure S10** Conceptual framework representing the association between genetically determined cannabis use and risk of schizophrenia 24

**Supplementary references** 25-26

**Supplementary analysis**

**The population-based risk of schizophrenia among users compared to non-users of cannabis**

The method to derive the odds ratio of genetically determined use of cannabis on risk of schizophrenia in the population is based on Ross *et al.*[^1^](#_ENREF_1)

Given:

**(A)** Prevalence of cannabis use in the European Union population = 0.133.[^2^](#_ENREF_2) This figure tallies with prevalence of ever use of cannabis in the Swedish cohort (=0.108),[^3^](#_ENREF_3) which thus allows comparison between the observational and causal estimates

**(B)** Prevalence of schizophrenia in non-users of cannabis = 0.006 (as retrieved from the Swedish cohort)[^3^](#_ENREF_3)

**(C)** Odds ratio for schizophrenia associated with genetically determined cannabis use (users vs. non users) at the population level

**(D)** Genetic association with schizophrenia as function of genetic association with use of cannabis (where causal genetic effects are expressed as Log OR per allele for both schizophrenia and use of cannabis)

The following can be calculated:

**(E)** Calculated population prevalence of schizophrenia: **(A × B × C) + (1 - A) × B**

**(F)** Estimated prevalence of schizophrenia in individuals with a theoretical increase in risk of use of cannabis of *e* = 2.72 fold: **(A × exp(1) × B × C) + (1 - A × exp(1)) × B**

**(G)** Estimated odds ratio for schizophrenia per *e* = 2.72 fold increase in risk of use of cannabis: **F/E**

It results that:

**(H)** $\exp\left( D \right)= G =\frac{F}{E}=\frac{\boldsymbol{(A \times exp(1) \times B \times C) + (1 - A \times exp(1)) \times B}}{\left( \boldsymbol{A \times B \times C} \right)\mathbf{+}\left( \mathbf{1 - A} \right)\boldsymbol{\times B}}$

As C is the only unknown variable, the association between genetically determined use of cannabis (cannabis users vs. non-users) and risk of schizophrenia (expressed as an odds ratio) at the population level can be calculated using algebraic transformations and (H) can be simplified into:

**(I)** $\mathbf{C=1+}\frac{\mathbf{(1-}\exp\left( \mathbf{D} \right)\mathbf{)}}{\left( \mathbf{ex}\mathbf{p} \left( \mathbf{D} \right)\mathbf{-}\exp\left( \mathbf{1} \right) \right)\boldsymbol{\times A}}$

**Proportion of the variance in use of cannabis**

The proportion of variance (conceptually similar to the R^2^) in use of cannabis was computed for each SNP based on the formula provided by Shim *et al*.:[^4^](#_ENREF_4)

$$R2=\frac{\text{2}\text{β}\text{2}\text{ }\text{x M}\text{AF}\text{ x }\text{(1}\text{ }\text{-}\text{ }\text{M}\text{AF}\text{)}}{\text{2}\text{β}\text{2}\text{ x M}\text{AF}\text{ x }\left( \text{1}\text{ }\text{-}\text{ }\text{M}\text{AF} \right)\text{ +}\text{ }\left( \text{se}\left( \text{β} \right) \right)\text{2}\text{ }\text{x 2}\text{N}\text{ x }\text{M}\text{AF}\text{ x }\text{(1}\text{ }\text{-}\text{ }\text{M}\text{AF}\text{)}}$$

with β, effect size (beta coefficient) for a given SNP, MAF, minor allele frequency, se(β), standard error of effect size, and *N*, sample size.

**UK Biobank: general description, genotyping, selection of participants and statistical analysis**

*General description*

The UK Biobank cohort is a large prospective cohort of 502,628 participants with phenotypic information, of whom around 152,249 have genetic information available as of July 2017 with the remainder due to be released in Q3 2016 .[^5^](#_ENREF_5) In the analysis, we reported on cross-sectional data at baseline. All participants attended one of 22 assessment centres from 2006 to 2010 where they completed a series of physical, sociodemographic, and medical assessments. Participants self-reported their smoking status as never, past or current. (A minority of n=299 refused to answer and these were removed).

*Genotyping*

UK Biobank genotyping was conducted by Affymetrix using a bespoke BiLEVE Axiom array for ~50,000 participants, and the remaining ~450,000 (for the purposes of this study 100,000) on a further updated bespoke Affymetric Axiom array (based on the 1st array). The two are extremely similar, sharing over 95% marker content. We controlled for array type as a covariate. Further information on the genotyping process is available on the UK Biobank website (<http://www.ukbiobank.ac.uk/scientists-3/genetic-data>), which includes detailed technical documentation (<http://www.ukbiobank.ac.uk/wp-content/uploads/2014/04/UKBiobank_genotyping_QC_documentation-web.pdf>). UK Biobank provide recommendations, which we followed, for which participants to exclude from analysis based on whether: the sample failed quality control; had significant missing data or heterozygosity. We used ten (UK Biobank provided) genetic principal components to account for population stratification. All SNPs present in the current analysis were in Hardy Weinberg equilibrium.

*Selection of participants and statistical analysis*

Of the 152,249 baseline participants, there were 112,197 participants who satisfied the inclusion criteria of: passed quality control; were Caucasian; had no first cousins or closer in the cohort, and had no mismatch between reported/genetically estimated sex and ethnicity. Of these 111,898 had smoking-related data. The mean age was 56.90 (SD = 7.93) years, and 53,122 (47.4%) were male. There were 59,914 never smokers and 51,984 ever smokers (46.46%).

Risk of ever smoking per SNP (0;1;2 dose model) was adjusted for age, sex, ten genetic principal components, batch, assessment centre and array.

All analyses were conducted with PLINK and STATA v.13.

**Table S1** Summary of the observational studies included in the analysis of cannabis use and risk of schizophrenia, presented by year of recruitment

| **Study** | **Full name (country)** | **Year(s) of recruitment** | **Years of follow-up** | **Cases** | **Controls** | **Exposure (as reported in the text)** | **Outcome (source)** | **Adjustments** | **Reference** |
| --- | --- | --- | --- | --- | --- | --- | --- | --- | --- |
| Swedish cohort | Cohort of Swedish conscripts (Sweden) | 1969-70 | 26 | 362 | 49,691 | Cannabis ever (use) | Schizophrenia (Swedish national hospital discharge) | Psychiatric diagnosis at conscription, IQ score, poor social integration, disturbed behaviour, cigarette smoking | [^3^](#_ENREF_3) |
| Dunedin | Dunedin Multidisciplinary Health and Development Study (New Zealand) | 1972-73 | 26 | 25 | 735 | Cannabis users by age 18 | Schizophreniform disorder (DSM-IV) | - | [^6^](#_ENREF_6) |
| ECA | Epidemiologic Catchment Area Program (US) | 1980-84 | 1 | 477 | 1,818 | Use of marijuana (vs. No use) | Self-reported psychotic experiences (Diagnostic Interview Schedule) | Sex, being in school, level of education, marital status, employment, presence at baseline of depressive episodes, manic episodes, agoraphobia and obsessive-compulsive disorder | [^7^](#_ENREF_7) |
| EDSP | Early Developmental Stages of Psychopathology Study (Germany) | 1995 | 4 | 424 | 2,013 | Any use (≥5 times) | Psychotic symptoms (Münich version of the composite international diagnostic interview [M-CIDI]) | Age, sex, socioeconomic status, living in city, childhood trauma, predisposition to psychosis at baseline, other drug use, tobacco, alcohol, predisposition for psychosis at follow-up and depression at baseline and follow-up | [^8^](#_ENREF_8) |
| NEMESIS | Netherlands Mental Health Survey and incidence Study (Netherlands) | 1996 | 3 | 38 | 4,007 | Baseline any use (of cannabis) | Any psychosis (based on the Brief Psychiatric Rating Scale) | Age, sex, ethnic group, level of education, unemployment and marital status, use of other drugs | [^9^](#_ENREF_9) |

**Table S2** Summary of the observational studies excluded from the analysis of cannabis use and risk of schizophrenia with reasons for exclusion presented by year of recruitment

| **Study** | **Full name/description (country)** | **Year(s) of recruitment** | **Years of follow-up** | **Exposure (definition)** | **Outcome (source)** | **Reason(s) for exclusion** | **Reference (PMID)** |
| --- | --- | --- | --- | --- | --- | --- | --- |
| CHDS | Christchurch Health and Development Study (New Zealand) | 1977 | 21 | Cannabis dependence (dependent vs. not dependent) based on *DSM-IV* diagnostic for cannabis dependence  and  frequency of cannabis use (from never use to daily use) | Psychotic symptoms (Symptom Checklist 90 [SCL-90] – 10 items) | Dependence severity (based on a count of *DSM-IV* cannabis dependence criteria) not available | Fergusson et al, Psychol Med, 2003 (12537032) and  Fergusson et al. Addiction, 2005 (15733249) |
| Zürich Study | Zürich Study (Switzerland) | 1978 | 30 | Frequency of cannabis use in adolescence (3 levels : none ; casual ; regular) | Schizophrenia nuclear symptoms subscale (SCL-90-R) | Lifetime use (ever vs. never users) not available | Rössler et al, Addiction, 2012 (22151745) |
| California | California inpatient hospital admissions | 1990-2000 | 10 | Any cannabis-related ICD-9 diagnostic code within a medical recode | Readmission with any schizophrenia diagnoses (ICD-9) | Use of inpatient data - hospitalization with any record of cannabis use and subsequent hospitalization for schizophrenia. | Callaghan et al., Am J Psychiatry, 2012 (22193527) |
| ALSPAC | Avon Longitudinal Study of Parents and Children | 1991-92 | 16 | Cumulative cannabis use at age 16 years (4 levels: never; 1-20 times; 21-60 times; >60 times) | Psychotic experiences (semi-structured interview based on PLIKSi) | Lifetime use (ever vs. never users) not available | Gage et al., Psychol Med, 2014 (25066001) |
| NPMS | British National Psychiatric Morbidity Survey | 2000 | 1.5 | Cannabis use (3 levels: not used in past year, used in past year but no report of dependence; dependence) corresponding to ever use of cannabis but only over the past one year  *and*  cannabis dependence (dependent vs. not dependent) | Psychotic symptoms (Psychosis Screening Questionnaire) | Lifetime use (ever vs. never users) and dependence severity (based on a count of *DSM-IV* cannabis dependence criteria) not available | Wiles et al., Br J Psychiatr, 2006 (16738341) |

**Table S3** Studies included in the cannabis-GWAS (Stringer *et al.*[^10^](#_ENREF_10))

| **Study** | **Country** | ***N total*** | **Reference/PMID** |
| --- | --- | --- | --- |
| *Discovery* | | | |
| ALSPAC | UK | 2976 | 22507743 |
| BLTS | Australia | 721 | 23187020 |
| CADD | USA | 853 | Not published |
| EGCUT1 | Estonia | 2765 | 15133739 |
| EGCUT2 | Estonia | 970 | 15133739 |
| FinnTwin | Finland | 1029 | 23298696 |
| HUVH | Spain | 981 | 25284319 |
| MCTFR | USA | 6241 | 23363460 |
| NTR | Netherlands | 4653 | 20477721 |
| QIMR | Australia | 6778 | 17988414 |
| TRAILS | Netherlands | 1226 | 18763693 |
| Utrecht | Netherlands | 1173 | 20925969 |
| Yale Penn European American | USA | 1964 | 24166409 |
|  | | | |
| *Replication* | | | |
| Radar | Dutch | 338 | 25466800 |
| SYS | Canada | 551 | 25454417 |
| TwinsUK | UK | 2078 | twinsuk.ac.uk |
| Yale Penn African American | US | 2660 | 24166409 |

ALSPAC, Avon Longitudinal Study of Parents and Children; BLTS, Brisbane Longitudinal Twin Study; CADD, Center on Antisocial Drug Dependence; EGCUT, Estonian Genome Center University of Tartu; FinnTwin, Finnish Twin Cohort (FinnTwin12 & FinnTwin16); HUVH, Hospital Universitari Vall d'Hebron; MCTFR, Minnesota Center for Twin and Family Research; NTR, Netherlands Twin Register; QIMR, Queensland Institute of Medical Research Berghofer adults; TRAILS, TRacking Adolescents’Individual Lives Survey; Utrecht, Utrecht Cannabis Cohort (CannabisQuest); Radar, Research on Adolescent Development and Relationships ; SYS, Saguenay Youth Study.

**Table S4** Summary of the 10 SNPs associated with use of cannabis (by increasing beta)

| **SNP** | **Chr** | **Position** | **EAF** | **Effect allele** | **Other allele** | **Sample size** | **Beta** | **SE** | **p-value** | **Number of studies directionally consistent**** |
| --- | --- | --- | --- | --- | --- | --- | --- | --- | --- | --- |
| rs35053471 | 3 | 47124761 | 0.62 | T | A | 31301 | 0.090 | 0.022 | 2.7 x 10^-6^ | 11/12 |
| rs12518098 | 5 | 60864467 | 0.68 | C | G | 32330 | 0.090 | 0.023 | 3.0 x 10^-6^ | 12/13 |
| rs4471463* | 11 | 112983595 | 0.45 | C | T | 32330 | 0.100 | 0.021 | 1.5 x 10^-6^ | 10/13 |
| rs4984460 | 15 | 96424399 | 0.25 | G | T | 32330 | 0.110 | 0.023 | 4.6 x 10^-7^ | 9/13 |
| rs7675351 | 4 | 141218757 | 0.14 | C | A | 24912 | 0.130 | 0.033 | 1.4 x 10^-6^ | 10/11 |
| rs73067624* | 1 | 196333461 | 0.10 | C | T | 24191 | 0.160 | 0.041 | 3.1 x 10^-6^ | 10/10 |
| rs2099149 | 12 | 30479358 | 0.19 | G | T | 22902 | 0.170 | 0.034 | 9.8 x 10^-7^ | 8/9 |
| rs2033867 | 2 | 175188281 | 0.06 | A | G | 22612 | 0.230 | 0.050 | 2.6 x 10^-6^ | 5/5 |
| rs58691539 | 2 | 52753909 | 0.09 | G | T | 12210 | 0.290 | 0.062 | 2.1 x 10^-6^ | 4/4 |
| rs7107977 | 11 | 915764 | 0.60 | A | G | 8759 | 0.290 | 0.064 | 1.9 x 10^-6^ | 6/6 |

* represent the two SNPs used in the sensitivity analysis and corresponding to two genes (*KCNT2* for rs73067624 and *NCAM1* for rs4471463), with a putative functional role, that were associated with ever use of cannabis in the gene-based tests of associations in ever use of cannabis-GWAS.[^10^](#_ENREF_10)

** represents directional consistency of the beta coefficients across studies under analysis.

None of the 10 SNPs were in linkage disequilibrium (R^2^<0.1) based on SNP Annotation and Proxy Search (SNAP, Broad Institute, MA, US).[^11^](#_ENREF_11)

Chr, chromosome; EAF, effect allele frequency; SE, standard error. Beta coefficient corresponds to log odds of ever use of cannabis.

**Table S5** Studies included in the Schizophrenia-GWAS (reproduced from the supplementary material of Ripke *et al.*[^12^](#_ENREF_12))

| **Study** | **Country** | **Cases** | **Controls** | **Reference/PMID** |
| --- | --- | --- | --- | --- |
| Umeå | Sweden | 341 | 577 | Not published |
| Umeå | Sweden | 193 | 704 | Not published |
| TOP | Norway | 377 | 403 | 19571808 |
| Uni. of Edinburgh | UK | 367 | 284 | 19571811 |
| Denmark | - | 876 | 871 | 19571808 |
| PEIC, WTCCC2 | Seven countries | 574 | 1812 | 23871474 |
| PEIC, WTCCC2 | Spain | 150 | 236 | 23871474 |
| New York, US & Israel | US & Israel | 325 | 139 | 20489179 |
| Ireland | Ireland | 264 | 839 | 19571811 |
| WTCCC2 | Ireland | 1291 | 1006 | 22883433 |
| GRAS | GRAS | 1067 | 1169 | 20819981 |
| EGCUT | Estonia | 234 | 1152 | 15133739 |
| EGCUT controls | Estonia | 347 | 310 | 15133739, 4166486 |
| EGCUT controls | Estonia | 636 | 636 | 15133739,24166486 |
| EGCUT controls | Estonia | 256 | 130 | 15133739,24166486 |
| EGCUT controls | Estonia | 1154 | 2310 | 15133739,24166486 |
| MGS | US, Australia | 2638 | 2482 | 19571809 |
| London | UK | 509 | 485 | 19571811 |
| Hubin | Sweden | 265 | 319 | 19571808 |
| Bulgaria | - | 195 | 608 | Not published |
| Toronto/Lilly (MIGen) | Canada & US | 526 | 1644 | Not published |
| Israel | - | 894 | 1594 | 24253340 |
| WTCCC controls | Six countries | 157 | 245 | 22885689 |
| New York | US | 190 | 190 | 17522711 |
| ASRB | Australia | 456 | 287 | 21034186 |
| Cardiff | UK | 396 | 284 | 19571811 |
| CLOZUK | UK | 3426 | 4085 | 22614287 |
| CLOZUK | UK | 2105 | 1975 | 22614287 |
| Netherlands | - | 700 | 607 | 19571808 |
| Finland | - | 186 | 929 | 19571808 |
| Finnish | - | 360 | 1082 | Not published |
| Portugal | - | 346 | 215 | 19571811 |
| CIDAR | US | 67 | 65 | 24424392 |
| Pfizer | - | 662 | 1172 | Not published |
| Bonn/Mannheim | Germany | 1773 | 2161 | 19571808 |
| Munich | Germany | 421 | 312 | 19571808 |
| Aberdeen | UK | 719 | 697 | 19571811 |
| CATIE | US | 397 | 203 | 18347602 |
| sw1 | Sweden | 215 | 210 | 23974872 |
| sw234 | Sweden | 1980 | 2274 | 23974872 |
| sw5 | Sweden | 1764 | 2581 | 23974872 |
| sw6 | Sweden | 975 | 1145 | 23974872 |
| CogUK | UK | 530 | 678 | 21850710 |
| NIMH CBDB | US | 133 | 269 | 11381111 |
| NIMH CBDB | US | 497 | 389 | 11381111 |
| Denmark | - | 471 | 456 | 19571808 |
| Bulgaria | - | 649 | 649 | 22083728 |
| Six countries | - | 516 | 516 | 22885689 |
| Bulgaria | - | 70 | 70 | Not published |
| Japan | - | 492 | 427 | 20832056 |
| STCRP | Singapore | 868 | 938 | Not published |
| China | - | 476 | 2018 | 24043878 |

For additional information on each study (incl. complete study name), see Ripke *et al.*[^12^](#_ENREF_12) Cases included individuals with schizophrenia or schizoaffective disorder. In addition, for some studies, cases were defined based on hospital discharge records, patients having treatment-resistant schizophrenia, or registered to use clozapine.

**Table S6** Power (two-sided α=0.05) for conventional Mendelian randomization analysis

| **Exposure** | **Actual N (Schizophrenia-GWAS)** | **Proportion of cases (Schizophrenia-GWAS)** | **Observational OR** | **R2 of instrument** | **N required for 80% power** | **Power at actual N** |
| --- | --- | --- | --- | --- | --- | --- |
| **Cannabis use** | 79,845 | 0.429 | 1.500* | 0.010 | 18,120 | 1.0 |

Power calculation was based on the method developed by Brion et al.[^13^](#_ENREF_13)

* from Swedish cohort.[^3^](#_ENREF_3)

Power at OR=1.37 (=causal estimate) is 0.99.

**Table S7** Comparison of conventional MR, MR-Egger, MR-Egger adjusted for SIMEX and weighted median MR causal effect estimates of cannabis use on risk of schizophrenia

| **Analysis** | **Causal effect estimate** | **95% CI** |
| --- | --- | --- |
| **Conventional MR** | 0.08 | 0.02; 0.13 |
| **MR-Egger** | 0.01 | -0.12; 0.14 |
| **MR-Egger+SIMEX** (*I^2^*=0.67) | 0.01 | -0.15; 0.16 |
| **Weighted median MR** | 0.05 | -0.02; 0.13 |

Estimates are expressed as Log OR per-1-log unit increase in ever use of cannabis. As previously described by Bowden et al,[^14^](#_ENREF_14) it is noteworthy to mention that power to detect a causal effect using MR-Egger analysis is largely underpowered (as shown by the corresponding large confidence intervals) with the use of 10 SNPs. The adjusted MR-Egger regression estimate (derived by simulation extrapolation [SIMEX] to account for a potential violation of the NOME assumption) is the result of 100,000 simulations.[^15^](#_ENREF_15) *I^2^* quantifies weak instrument bias in the context of MR-Egger.

**Figure S1** Flowchart of observational meta-analysis


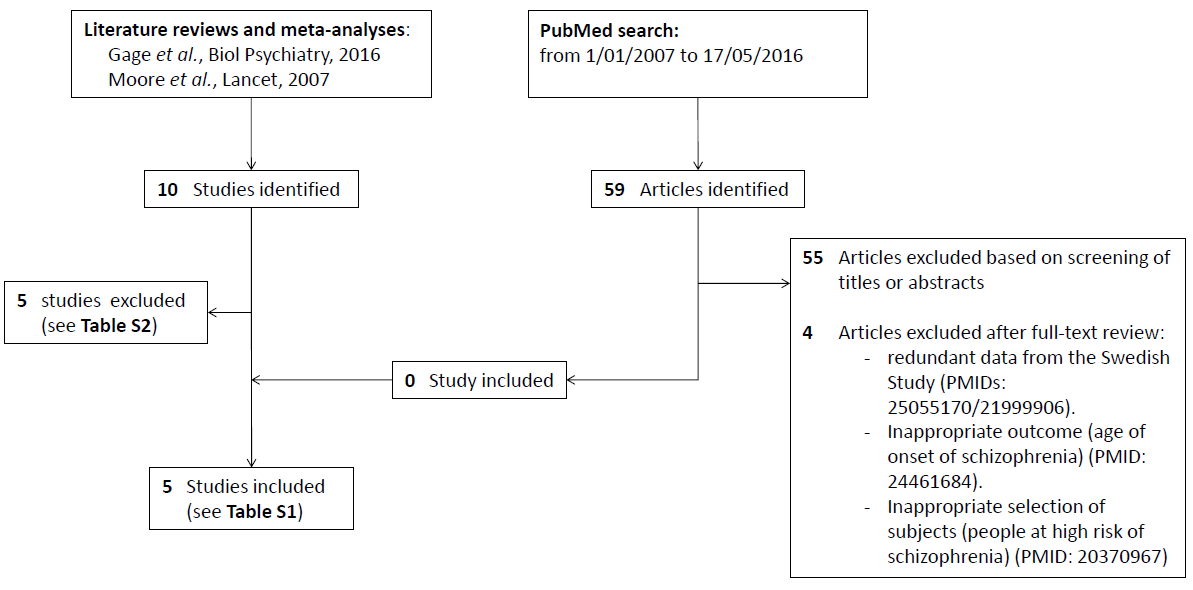


PubMed Search was conducted on 17 May 2016 to retrieve additional prospective observational studies reporting an association between cannabis use and risk of schizophrenia since the last meta-analysis on the subject published in 2007. PubMed terms applied for the search: ("cannabis"[MeSH Terms] OR "marijuana"[MeSH Terms]) AND "schizophrenia"[MeSH Terms] AND (("2007/01/01"[PDAT] : "2016/12/31"[PDAT]) AND "humans"[MeSH Terms]).

**Figure S2** Venn diagram showing the number of individuals and the overlap between the cannabis-GWAS and the schizophrenia-GWAS


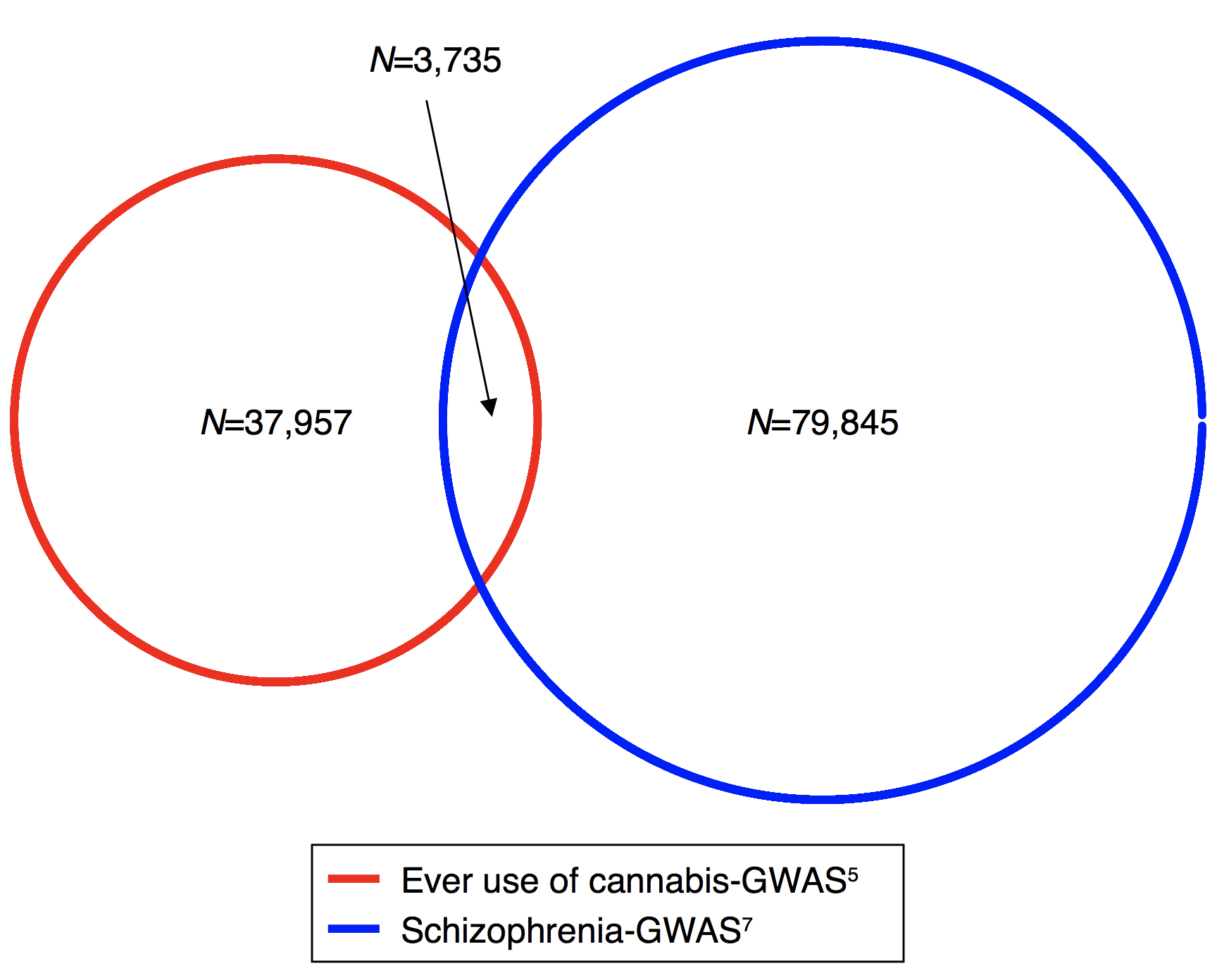


Based on the data provided in Stringer *et al.*[^10^](#_ENREF_10) and Ripke *et al.*[^12^](#_ENREF_12), only the EGCUT study (Estonian Genome Center University of Tartu (*N*=3,735) – see **Tables S3 & S5**) contributed to both GWAS.

**Figure S3** Pair-wise association plot of the 10 SNPs associated with cannabis use and risk of schizophrenia


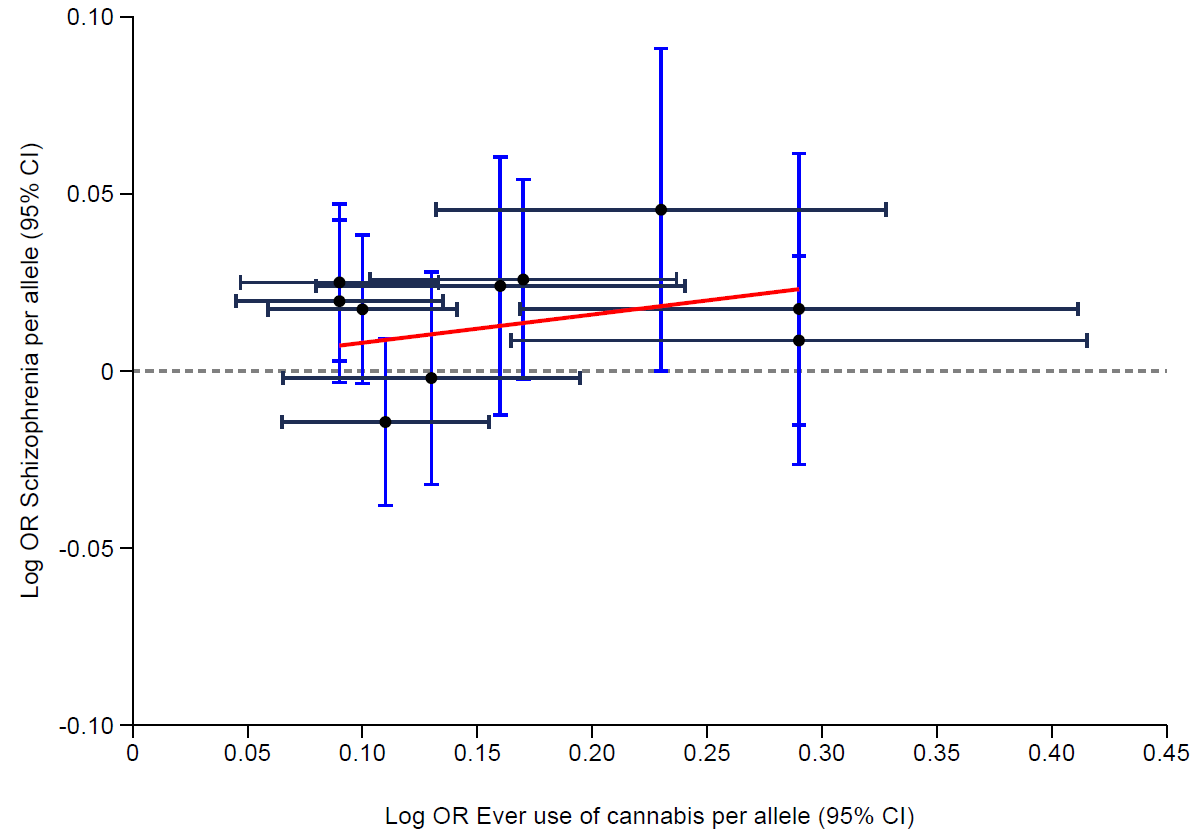


The red line represents the regression slope of the causal effects estimates (derived by the inverse-variance weighted approach as proposed by Bowden *et al.*).[^14^](#_ENREF_14)

**Figure S4** Scatter plot of the genetic association with cannabis use against genetic association with schizophrenia


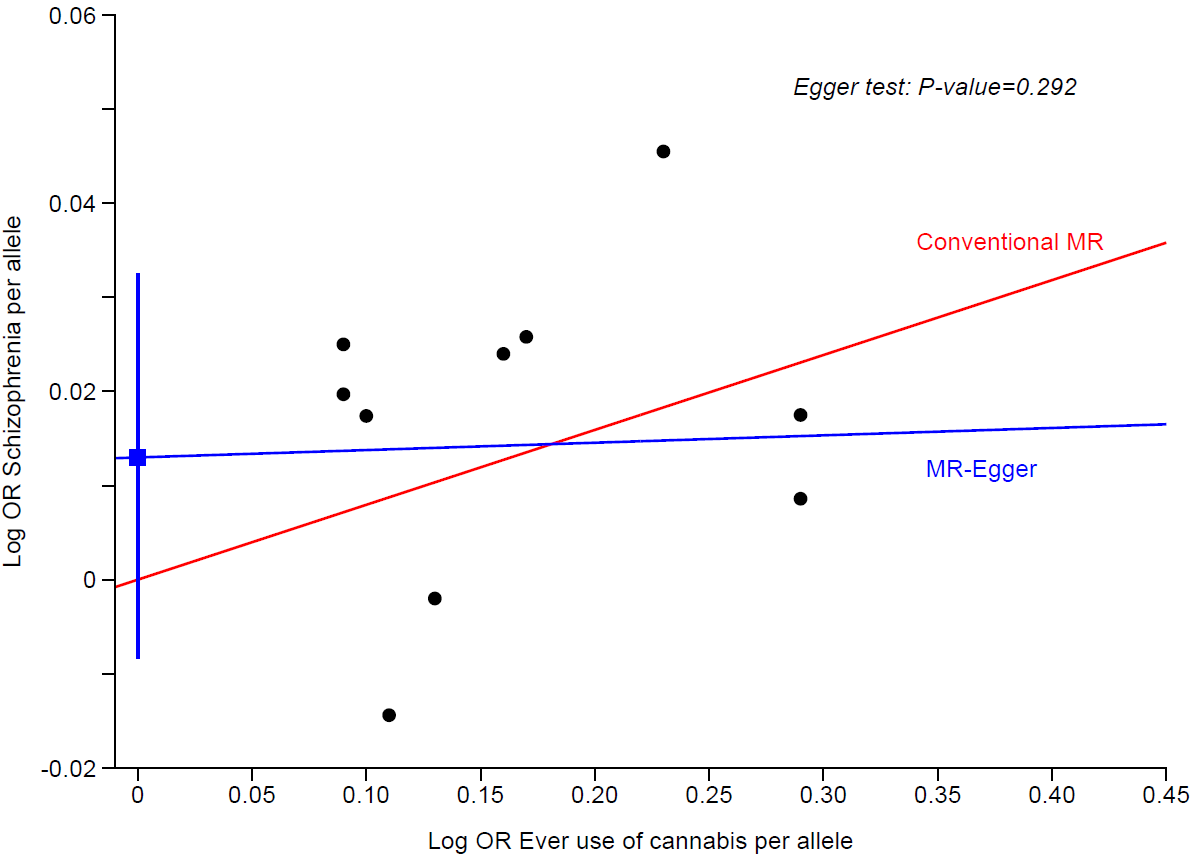


The conventional Mendelian randomization (Conventional MR in red) and Egger Mendelian randomization (MR-Egger in blue) causal effects estimates are presented as regression slopes. The constant and its 95% CI (obtained by bootstrap resampling 10,000 times) derived from Egger regression are shown as the blue square and vertical bar, respectively.

**Figure S5** Funnel plot of the instrument strength (minor allele frequency corrected genetic association with cannabis use) against causal estimates of cannabis use on schizophrenia


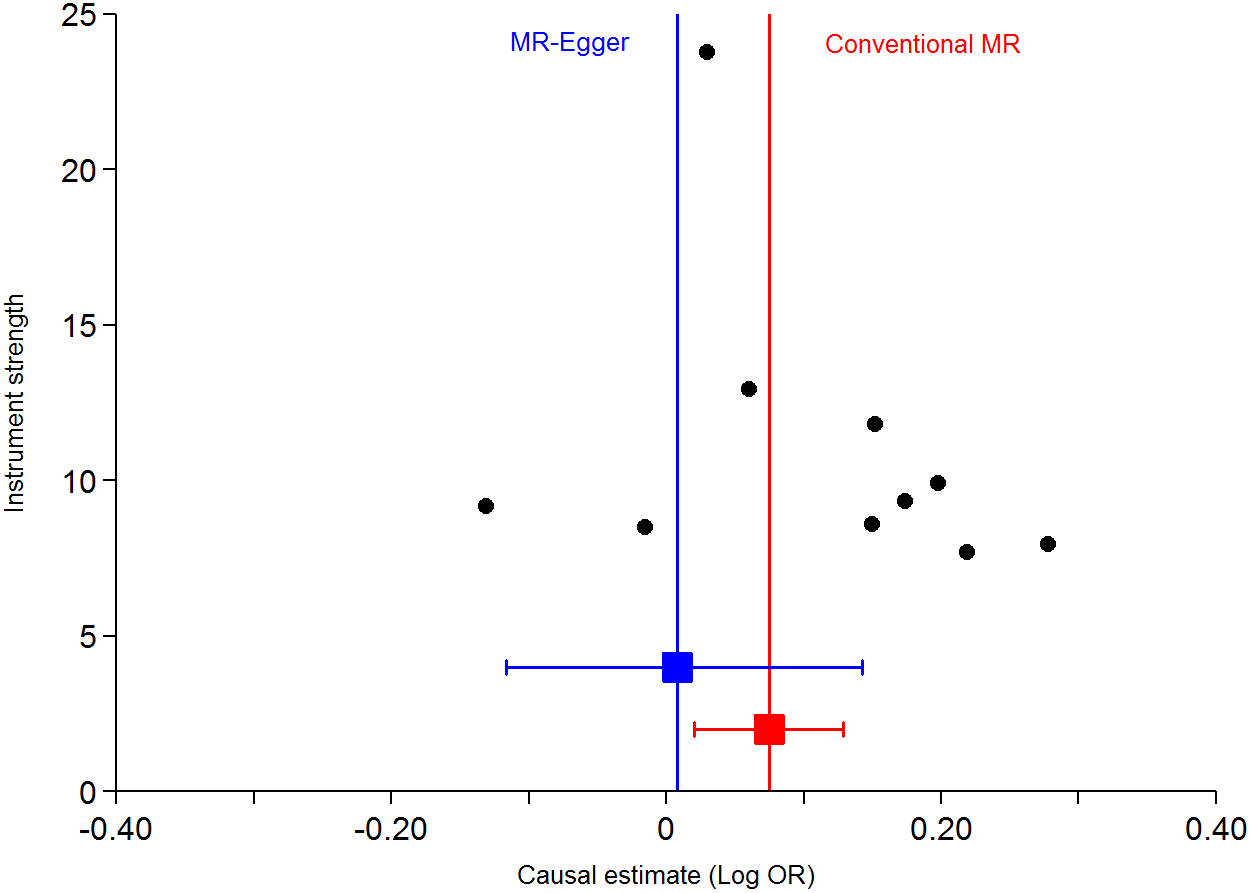


The instrument strength, representing the minor allele frequency corrected genetic association with ever use of cannabis is calculated by dividing the SNP-exposure association by the standard error of the SNP-outcome association for each SNP.[^14^](#_ENREF_14) The conventional Mendelian randomization (Conventional MR in red) and Egger Mendelian randomization (MR-Egger in blue) causal effect estimates are presented.

**Figure S6** Studentized residuals of conventional MR analysis


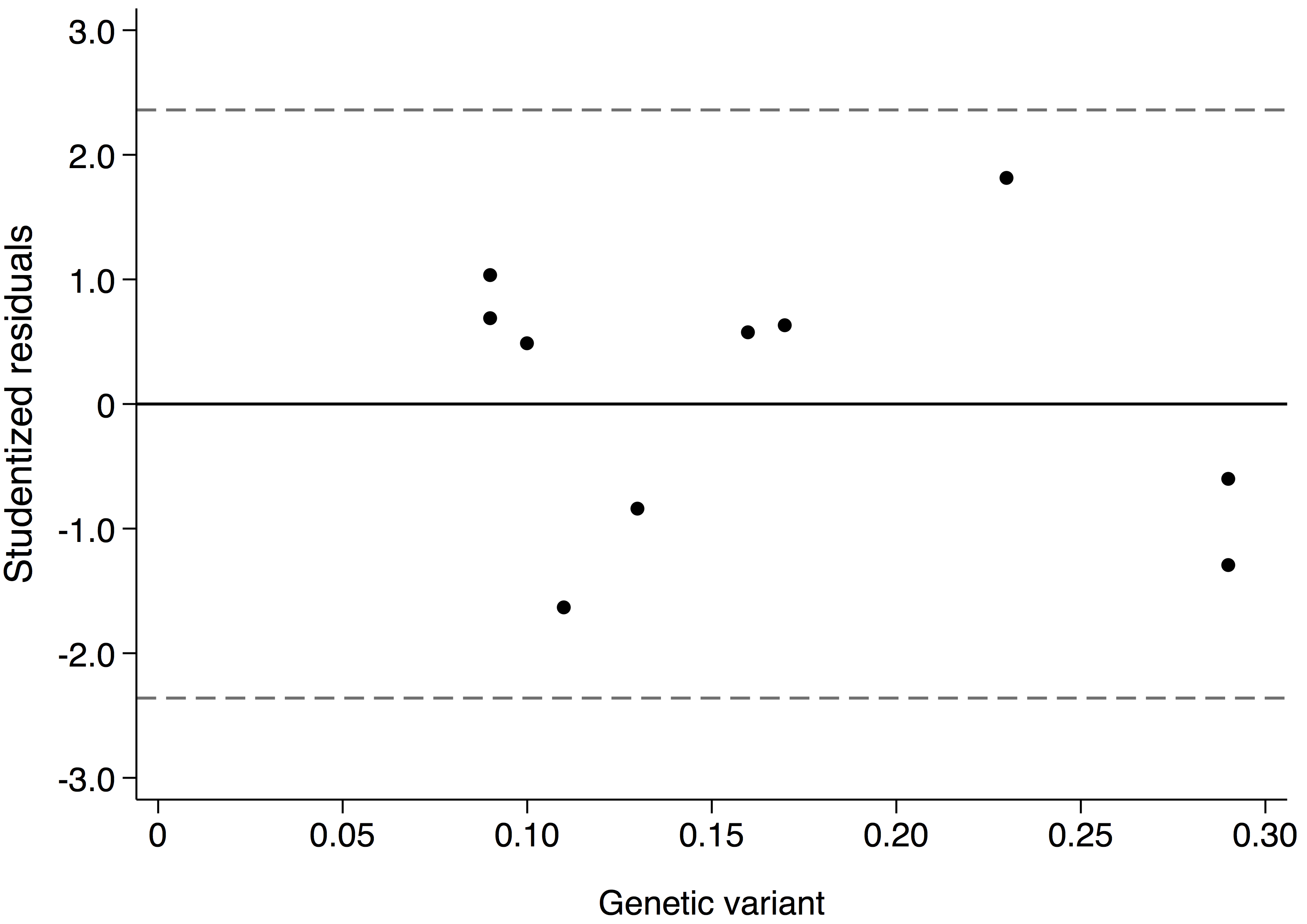


The studentized residuals were derived from a linear regression model based on the inverse-variance weighted approach (regression of SNP-cannabis estimates on SNP-schizophrenia estimates weighted by the minor allele frequency) as proposed by Bowden et al.[^14^](#_ENREF_14) Dashed grey lines represent the range where 95% of the studentized residuals should lie if the model is correct.

**Figure S7** Cook’s distance of conventional MR analysis

**
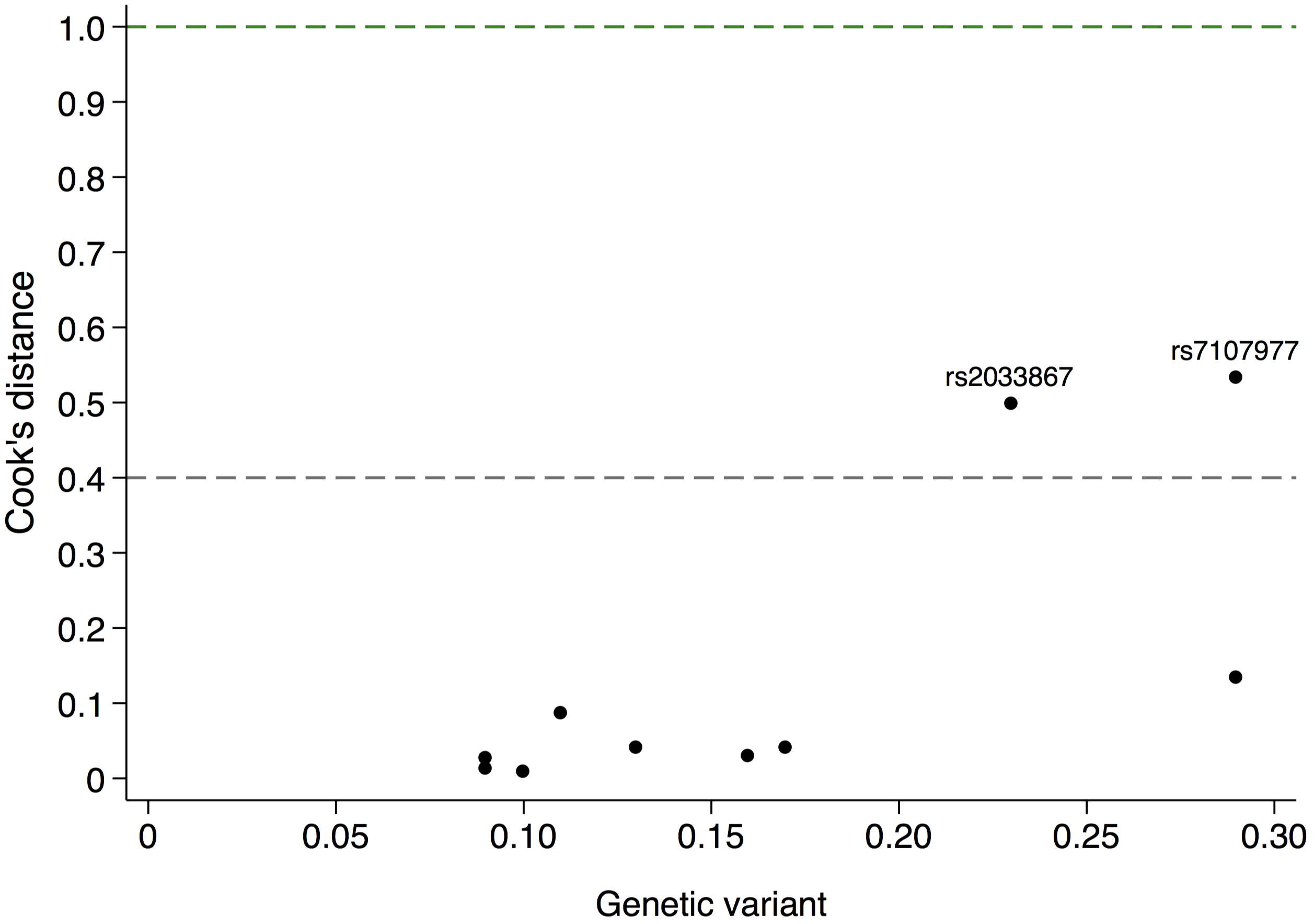
**

Originally Cook proposed that values of distance greater than one identify influential observations (dashed green line).[^16^](#_ENREF_16) Hamilton proposed a more stringent approach by investigating observations whose Cook’s distance is greater than 4/n (here, n=10) (dashed grey line).[^17^](#_ENREF_17)

**Figure S8** Contrasting main results with analysis removing two SNPs with potential influential level on the model based on Cook’s distance

**
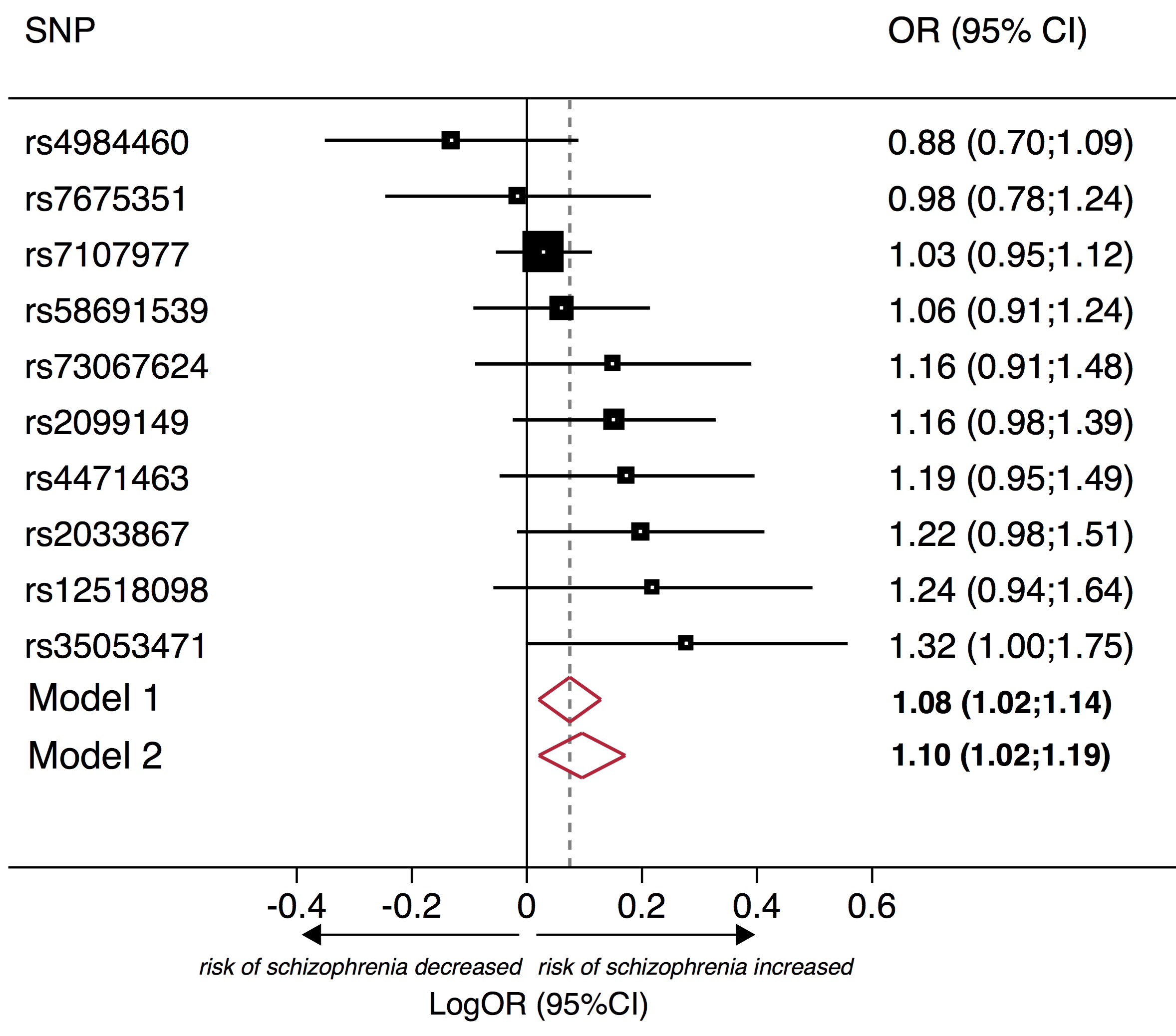
**

Fixed-effect meta-analysis of the causal estimates of use of cannabis-SNPs on risk of schizophrenia using the ten SNPs from the main analysis (model 1) and after removal of rs2033867 and rs7107977 (model 2) (see **Figure S7**). Log odds ratios (Log OR) (plotted) and OR (tabulated) (±95% CI) express the risk of schizophrenia per-1-log unit increase in ever use of cannabis.

**Figure S9** Sensitivity analyses of the association of cannabis use and risk of schizophrenia restricting to two SNPs with putative functional roles


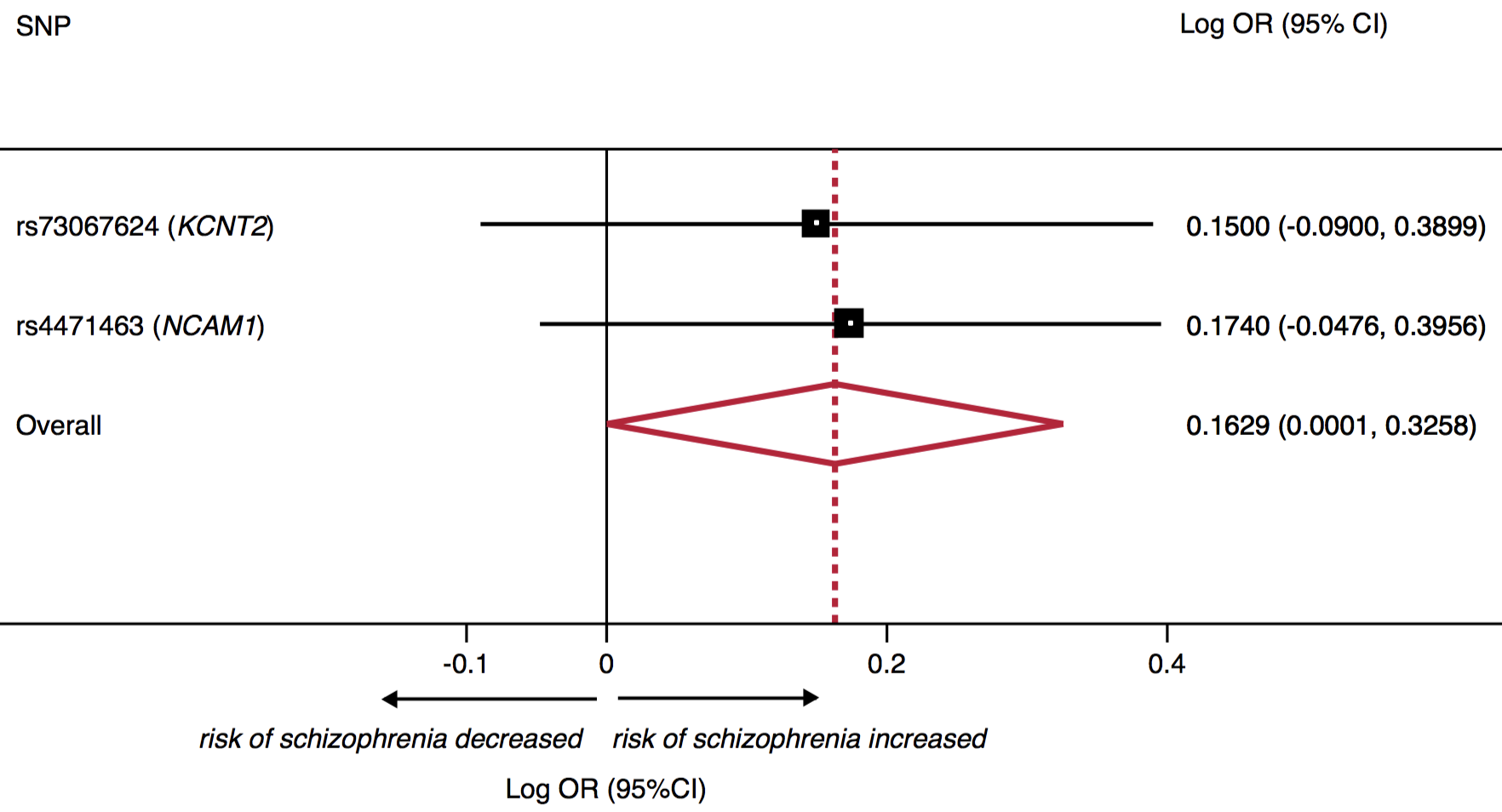


Fixed-effect meta-analysis of the causal estimates of use of cannabis-SNPs. Genes with presumptive function roles are indicated in brackets. Log odds ratios (Log OR) and 95% confidence intervals (CI) express the risk of schizophrenia per-1-log unit increase in ever use of cannabis and the corresponding combined OR (95% CI) is 1.18 (1.00-1.39). The method to derive the population-based OR of schizophrenia (OR 1.88; 95%CI, 1.00-3.21) among users of cannabis compared to non-users, as presented in the main text, is described on **pages 3-4** of the **Supplement**.

**Figure S10** Conceptual framework representing the association between genetically determined cannabis use and risk of schizophrenia


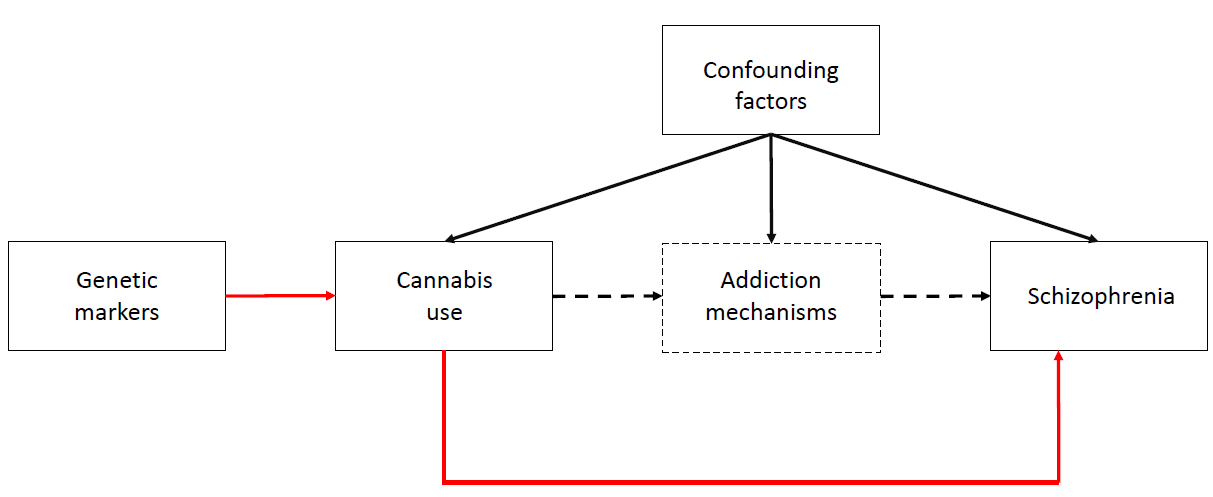


The findings of our Mendelian randomization analysis – in line with previous randomized trials in laboratory conditions on cannabis and psychotic symptoms - support the pathway indicated with the solid red lines, where cannabis use causally influences the risk of schizophrenia. The use of genetic markers diminishes the possibility that confounding factors (e.g. demographic, parental history, personal history, socioeconomic or other environmental confounders) are explaining the association that we report (solid black lines). It is nonetheless possible that genetic markers associate with schizophrenia through different pathway(s), but this is not supported by the results of Egger Mendelian randomization that showed absence of unmeasured pleiotropy of the genetic markers employed. Finally, it is possible that genetically determined cannabis use influences addiction mechanisms which in turn determine the risk of schizophrenia (dashed black lines). Supported by our sensitivity analysis restricting on two genetic markers potentially associated with addiction, this hypothesis still represents a valid interpretation of the results, as cannabis use – which triggers addiction - would then lie on the causal pathway to schizophrenia.

**Supplementary references**

1. Ross S, Gerstein HC, Eikelboom J, Anand SS, Yusuf S, Pare G. Mendelian randomization analysis supports the causal role of dysglycaemia and diabetes in the risk of coronary artery disease. *Eur Heart J* 2015; **36**: 1454-1462.

2. European Monitoring Centre for Drugs and Drug Addiction (2016), European Drug Report 2016: Trends and Developments, Publications Office of the European Union, Luxembourg. (<http://www.emcdda.europa.eu/system/files/publications/2637/TDAT16001ENN.pdf>). Accessed 2016, Aug 06.

3. Zammit S, Allebeck P, Andreasson S, Lundberg I, Lewis G. Self reported cannabis use as a risk factor for schizophrenia in Swedish conscripts of 1969: historical cohort study. *BMJ* 2002; **325**: 1199.

4. Shim H, Chasman DI, Smith JD, et al. A multivariate genome-wide association analysis of 10 LDL subfractions, and their response to statin treatment, in 1868 Caucasians. *PloS ONE* 2015; **10**. doi:10.1371/journal.pone.0120758.

5. Sudlow C, Gallacher J, Allen N, et al. UK biobank: an open access resource for identifying the causes of a wide range of complex diseases of middle and old age. *PLoS Med* 2015; **12**. doi:10.1371/journal.pmed.1001779.

6. Arseneault L, Cannon M, Poulton R, Murray R, Caspi A, Moffitt TE. Cannabis use in adolescence and risk for adult psychosis: longitudinal prospective study. *BMJ* 2002; **325**: 1212-1213.

7. Tien AY, Anthony JC. Epidemiological analysis of alcohol and drug use as risk factors for psychotic experiences. *J Nerv Ment Dis* 1990; **178**: 473-480.

8. Henquet C, Krabbendam L, Spauwen J, et al. Prospective cohort study of cannabis use, predisposition for psychosis, and psychotic symptoms in young people. *BMJ* 2005; **330**. doi 10.1136/bmj.38267.664086.63.

9. van Os J, Bak M, Hanssen M, Bijl RV, de Graaf R, Verdoux H. Cannabis use and psychosis: a longitudinal population-based study. *Am J Epidemiol* 2002; **156**: 319-327.

10. Stringer S, Minica CC, Verweij KJ, et al. Genome-wide association study of lifetime cannabis use based on a large meta-analytic sample of 32 330 subjects from the International Cannabis Consortium. *Transl Psychiatry* 2016; **29**: 36. doi: 10.1038/tp.2016.

11. Johnson AD, Handsaker RE, Pulit SL, Nizzari MM, O'Donnell CJ, de Bakker PI. SNAP: a web-based tool for identification and annotation of proxy SNPs using HapMap. *Bioinformatics* 2008; **24**: 2938-9. (<http://www.broadinstitute.org/mpg/snap/ldsearch.php>).

12. Schizophrenia Working Group of the Psychiatric Genomics Consortium. Biological insights from 108 schizophrenia-associated genetic loci. *Nature* 2014; **511**: 421-427.

13. Brion MJ, Shakhbazov K, Visscher PM. Calculating statistical power in Mendelian randomization studies. *Int J Epidemiol* 2013; **42**: 1497-1501.

14. Bowden J, Davey Smith G, Burgess S. Mendelian randomization with invalid instruments: effect estimation and bias detection through Egger regression. *Int J Epidemiol* 2015; **44**: 512-525.

15. Bowden J, Del Greco MF, Minelli C, Davey Smith G, Sheehan NA, Thompson JR. Assessing the suitability of summary data for two-sample Mendelian randomization analyses using MR-Egger regression: the role of the I2 statistic. *Int J Epidemiol* 2016. doi: 10.1093/ije/dyw220. [Epub ahead of print].

16. Cook RD. Detection of influential observation in linear regression. *Technometrics* 1977; **19**: 15–18.

17. Hamilton LC. Regression with Graphics: A Second Course in Applied Statistics. 1992. Belmont, CA: Duxbury.
